# Work-life realities of doctors in Georgia and their impact on mental health and work-related well-being: A cross-sectional study

**DOI:** 10.1371/journal.pone.0349641

**Published:** 2026-05-29

**Authors:** Elene Asanidze, Zezva Asanidze, Revaz Lordkipanidze, Manana Tsertsvadze, Aleksandre Asanidze, Nino Parunashvili, Jenaro Kristesashvili, Ana Jibladze, Swarali Yatin Chodnekar, Besik Asanidze

**Affiliations:** 1 Petre Shotadze Tbilisi Medical Academy, Tbilisi, Georgia; 2 Institute of Ecology, Ilia State University Tbilisi, Tbilisi, Georgia; 3 Tbilisi State Medical University, Tbilisi, Georgia; 4 University of Toronto, Physician Assistant Program, Toronto, Canada; 5 Ivane Javakhishvili Tbilisi State University, Tbilisi, Georgia; King Abdulaziz University Faculty of Medicine, SAUDI ARABIA

## Abstract

**Background:**

Physicians worldwide are exposed to high occupational demands, long working hours, and emotional strain, placing them at increased risk for stress, depressive symptoms, and impaired occupational well-being. However, data describing these challenges among physicians in Georgia remain limited.

**Study objective:**

This study aimed to assess the prevalence of stress and depressive symptoms among physicians in Georgia across medical specialties and to examine their associations with selected work-related and psychosocial well-being indicators, including emotional well-being, work satisfaction, sleep-related factors, and future-oriented optimism, with attention to differences by specialty, gender, and professional experience.

**Materials and methods:**

A cross-sectional study was conducted using an anonymous, structured online survey administered between February 5 and May 25, 2024, in Tbilisi, Georgia. A non-probability convenience sampling method was employed. Participation was voluntary, and inclusion criteria required respondents to be 25 years or older and currently employed in either a medical or non-medical profession in Georgia. The study included 390 physicians and a control group of 240 non-medical professionals. Physicians were categorized by medical specialty. Stress and depression were assessed using the Depression, Anxiety, and Stress Scale–21 (DASS-21) and the Patient Health Questionnaire–9 (PHQ-9). Both the PHQ-9 and the DASS-21 depression subscale were included to capture depressive symptom severity using complementary, widely used screening instruments, with analyses focusing on consistency of patterns rather than direct score equivalence. Additional items captured demographic characteristics and work-related and psychosocial factors. Data were collected electronically via Microsoft Forms and analyzed using appropriate statistical methods. Analyses were primarily descriptive and bivariate.

**Results:**

In unadjusted analyses, physicians reported significantly higher levels of stress and moderate-to-severe depression compared with non-medical participants (*p* < 0.05). Stress levels decreased with increasing years of professional experience (*p* < 0.001) and were higher among female physicians (OR = 1.7), whereas depression was more prevalent among male physicians (OR = 2.5). Married physicians reported higher stress levels than unmarried physicians (*p* < 0.05). Obstetrics–gynecology, general surgery, urology, and anesthesiology exhibited the highest prevalence of stress and depression (*p* < 0.05). Work-related stressors, sleep disturbances, and reduced optimism were more common among younger physicians.

**Conclusion:**

Physicians in Georgia reported significantly higher levels of stress and depressive symptoms compared with non-medical professionals, with potential implications for occupational well-being and healthcare system sustainability. These findings may suggest the relevance of targeted, specialty-sensitive approaches addressing workload, organizational factors, and psychosocial support to promote physician well-being and support healthcare quality in Georgia.

## Introduction

The role of a physician in society is defined not only by the critical nature of medical practice and adherence to high professional and ethical standards, but also by significant personal sacrifices made in the service of patient care [1–3]. The inherently demanding nature of medical work requires sustained emotional, cognitive, and physical engagement, often leading to prolonged working hours, high responsibility, and limited opportunities for rest and personal life. As a result, physicians frequently experience elevated levels of stress, overwork, and neglect of personal health, family relationships, and social well-being [2–4].

Stress is defined as the body’s nonspecific response to demands requiring adaptation to environmental challenges [3–5]. In medical practice, stress commonly arises from complex clinical decision-making, high patient expectations, time pressure, and exposure to human suffering. While physicians routinely manage acute stressors, the cumulative burden of persistent workplace pressures can lead to chronic stress, contributing to emotional, mental, and physical exhaustion, often conceptualized as burnout syndrome [3–6]. Burnout has been widely recognized as a consequence of sustained occupational stress and impaired coping mechanisms and represents a major challenge within healthcare systems globally [3–7].

Chronic stress and emotional exhaustion among healthcare professionals are closely associated with depressive symptoms [8–11]. Depression is a common mental health disorder characterized by persistent low mood, loss of interest or pleasure, cognitive impairment, and somatic symptoms that interfere with daily functioning [8–10]. Among physicians, depression not only diminishes individual perceived well-being but may also negatively affect professional performance, patient safety, and the overall quality of healthcare delivery. Understanding depression within the healthcare workforce is therefore essential for promoting both clinician well-being and optimal patient outcomes.

In Georgia, these challenges are further compounded by systemic and socioeconomic factors shaping the healthcare environment. Ongoing workforce shortages in certain specialties, uneven distribution of healthcare resources, and high patient volumes place substantial demands on physicians. In addition, relatively low remuneration and financial insecurity often require physicians to work across multiple hospitals or healthcare facilities to maintain adequate income. This multi-site employment frequently results in extended working hours, reduced opportunities for rest and recovery, and limited access to consistent institutional support. Together, these structural constraints may intensify occupational stress and increase vulnerability to adverse mental health outcomes, positioning physician well-being as a broader public health and societal concern rather than solely an individual issue.

Although quality of life is frequently discussed in relation to physician well-being, it broadly refers to individuals’ perceptions of physical, psychological, and social functioning within their cultural and value contexts [10–12]. In occupational health research, work-related and psychosocial factors, such as job satisfaction, work–life balance, sleep quality, and future professional expectations, are commonly examined as indicators of occupational and psychosocial well-being that reflect how stress and depression affect daily functioning [5–12]. These dimensions are particularly relevant in healthcare settings, where impaired well-being among physicians may influence workforce sustainability and quality of care.

Identifying and addressing these stressors is therefore critical to developing strategies that mitigate their impact on healthcare professionals and support the functioning of healthcare systems.

Recent international studies indicate that physician mental health is a growing concern, with burnout prevalence estimates ranging from 45% to 55% in many countries [8–12]. Although the global literature on physician stress and depression is extensive, findings vary across healthcare systems and sociocultural contexts [8–13]. In Georgia, comprehensive data examining stress, depression, and associated work-related and psychosocial well-being indicators across medical specialties remain limited. This lack of evidence highlights the need for context-specific research to better understand the mental health challenges faced by physicians in Georgia and to inform targeted interventions aimed at improving physician well-being and healthcare quality.

### Study objective

To assess the prevalence of stress and depressive symptoms among physicians in Georgia across medical specialties and to examine their associations with selected work-related and psychosocial well-being indicators, including emotional well-being, work satisfaction, sleep-related factors, and future-oriented optimism, with attention to differences by specialty, gender, and professional experience.

## Materials and methods

This study employed a cross-sectional design using an anonymous, structured online survey to assess stress, depression, and selected work-related and psychosocial well-being indicators among physicians in Georgia. The survey was conducted from February 5 to May 25, 2024, in Tbilisi, Georgia.

The study used a non-probability convenience sampling method. Participants were recruited through institutional mailing lists, medical associations, and hospitals for healthcare professionals, while non-medical participants were reached through social media platforms and public networks to access a broader sample of the general population. Participation was voluntary, and inclusion criteria required that respondents be aged 25 years or older and currently employed in either a medical or non-medical profession in Georgia.

Given the use of non-probability convenience sampling and different recruitment channels, the non-medical group was included for contextual comparison rather than population-level inference. Accordingly, interpretation emphasizes within-physician analyses, with comparisons to the non-medical group presented descriptively.

The study included 390 physicians and a control group of 240 non-medical professionals. Physicians were further divided into subgroups based on their specialties, including obstetrics–gynecology, reproductive medicine, anesthesiology, urology, general surgery, traumatology, neurosurgery, neurology, endocrinology, and radiology. The control group consisted of individuals working in non-medical fields such as education, law, business, engineering, and information technology, who self-identified as non-medical professionals in the survey.

This study was planned as an exploratory, hypothesis-generating investigation rather than a hypothesis-testing study. All analyses were unadjusted and should be interpreted as descriptive and exploratory. Because of the cross-sectional design, non-probability convenience sampling, and differences in recruitment between the physician and non-medical groups, the study was not designed to support causal inference or independent effect estimation.

### Measures

Stress and depressive symptoms were assessed using two widely recognized questionnaires adapted for the Georgian context: the Depression, Anxiety, and Stress Scale–21 (DASS-21) and the Patient Health Questionnaire–9 (PHQ-9) [13–15]. Both the PHQ-9 and the DASS-21 depression subscale were included to capture depressive symptom severity using complementary, widely used screening instruments. Analyses focused on consistency of patterns rather than direct score equivalence, and the instruments were not treated as interchangeable diagnostic tools. The DASS-21 assesses symptoms of depression, anxiety, and stress using established cut-off scores for each domain, while the PHQ-9 evaluates the frequency of depressive symptoms, with scores of 9 or higher indicating clinically significant depressive symptoms.

To adapt the DASS-21 and PHQ-9 for the Georgian context, a standardized process of translation and cultural adaptation was followed. The original instruments were translated into Georgian by bilingual experts and then back-translated into English to assess semantic equivalence. The translated versions were pre-tested on a small sample of physicians to evaluate clarity, relevance, and contextual appropriateness prior to use in the main survey. However, this process did not constitute a full formal psychometric validation study in the Georgian physician population.

Internal consistency reliability for the study sample was assessed using Cronbach’s alpha coefficients. In the current study sample, the Georgian version of the PHQ-9 and the DASS-21 subscales demonstrated acceptable to good internal consistency, with Cronbach’s alpha coefficients meeting or exceeding the commonly used threshold of α ≥ 0.80. These estimates are sample-specific and derived from the current study population.

### Demographic and work-related variables

Demographic characteristics collected included age, gender, marital status, medical specialty, and years of professional experience. These data were obtained through direct questions in the first section of the survey.

To investigate environmental and systemic contributors to stress and depression, the survey included additional items related to workplace and personal stressors. These questions were designed to assess the perceived impact of various factors, with responses ranked to reflect the degree of influence. Items included the number of night shifts per week, average sleep duration, perceived workload, frequency of professional conflicts, and perceived work–life balance. All items were assessed using structured multiple-choice questions developed by the research team.

Based on relevance to the study objectives, the following variables were retained for analysis: work intensity, job satisfaction, satisfaction with the work environment, remuneration satisfaction, and optimism regarding future professional expectations.

Data collection was conducted electronically via Microsoft Forms, with participants completing a structured online survey. The survey included standardized questionnaires and closed-ended questions related to stress levels, depressive symptoms, and work-related and psychosocial indicators.

### Statistical analysis

Statistical analyses were primarily descriptive and bivariate. Continuous variables across groups were compared using Student’s t-test, with Welch’s correction applied where variances were unequal. Pearson’s correlation coefficient (r) was used for correlation analyses. Statistical significance was established at p < 0.05 for all analyses, conducted using SPSS version 25 [16]. No multivariable adjustment was performed; therefore, the reported associations are unadjusted and may be influenced by confounding. Accordingly, comparisons between physicians and non-medical participants, as well as subgroup analyses by specialty, sex, marital status, and professional experience, should be interpreted cautiously.

Physicians within each specialty were categorized into two groups based on depression severity: low depression (no or mild depression) and high depression (moderate or severe depression). Odds ratios (ORs) were calculated as the ratio of physicians with high depression to those with low depression within each specialty.

Ninety-five percent confidence intervals (95% CIs) for odds ratios were calculated using standard methods.

### Sample size and power considerations

A formal a priori sample size calculation was not performed due to the exploratory nature of the study and the use of a non-probability convenience sampling approach. The achieved sample size allowed descriptive comparisons across the included groups; however, subgroup findings should be interpreted cautiously, particularly where the number of participants within specialties may have been limited. With 390 physicians and 240 non-medical participants, the study had sufficient statistical power (>80%) to detect small-to-moderate effect size differences (Cohen’s *d* ≥ 0.3) in stress and depression outcomes between groups at a two-sided significance level of 0.05. The sample size was also adequate for subgroup analyses by medical specialty, gender, and years of professional experience.

### Conflict of interest

The authors declare no conflict of interest in preparing this article.

### Ethical considerations

The study protocol was reviewed and approved by the Ethics Committee of the Reproductive Medicine Center “Universe” (Approval No. 2/2023). The survey was anonymous, and no personally identifiable information was collected. Prior to accessing the questionnaire, participants were provided with detailed information regarding the study objectives, voluntary nature of participation, and confidentiality of responses. Electronic informed consent was obtained through an online consent statement, and participants proceeded to the survey only after indicating their agreement. Given the anonymous design and minimal risk to participants, no written or oral consent was required. Participants were free to discontinue the survey at any point without providing a reason.

## Results

### Participant characteristics

**Fig 1:**
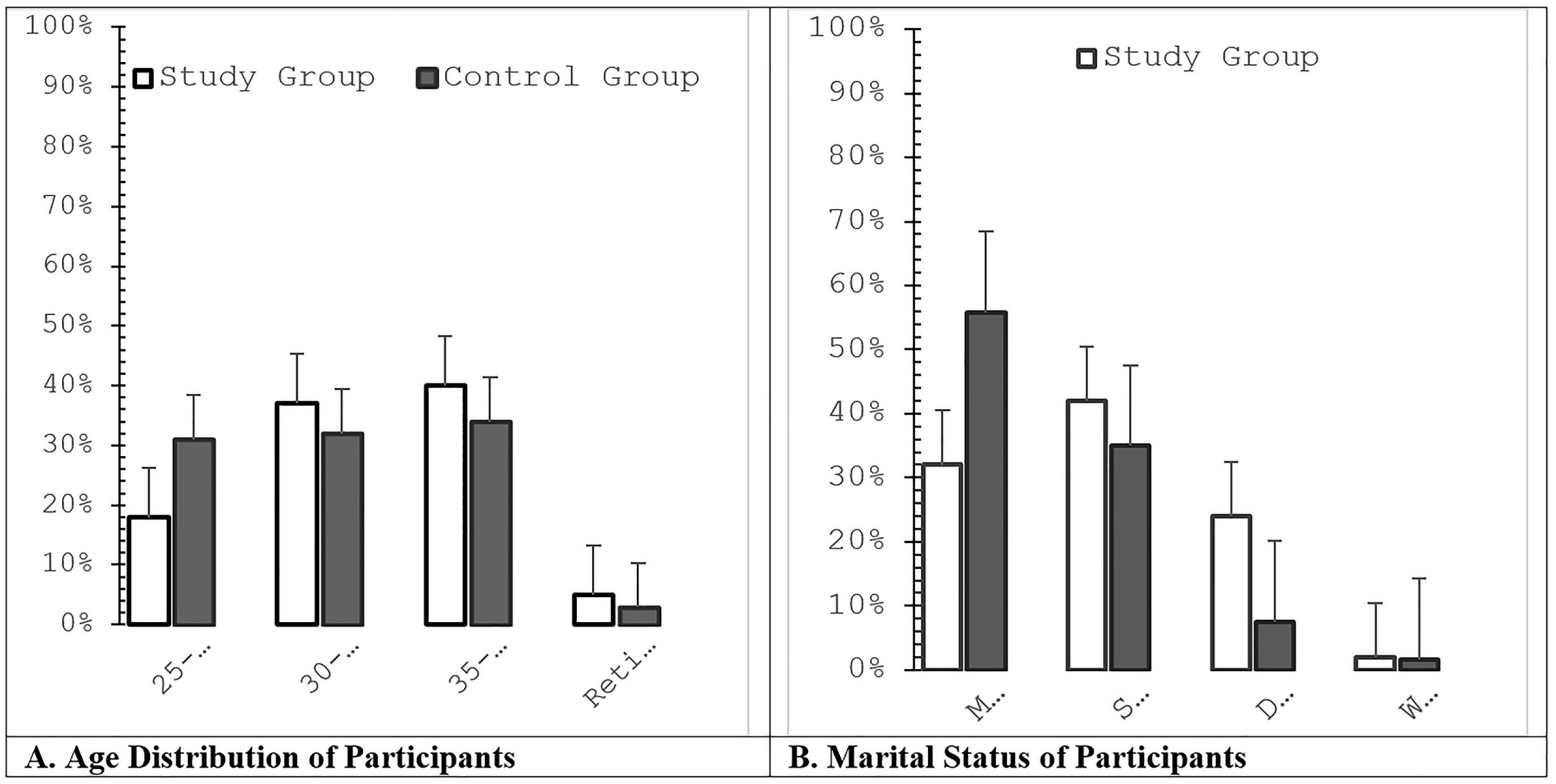
Descriptive statistics of participants in the study and control groups.

The study included 390 physicians and 240 non-medical professionals. The mean age of physicians was 42 ± 7.4 years, compared with 38 ± 6.5 years among non-medical participants, with no statistically significant difference between groups. Participants aged 25–30 years were more common in the non-medical group, while no significant differences were observed across other age categories (Fig 1A).

Significant differences were observed in marital status distribution between groups (*p* < 0.05). Single and divorced individuals were more prevalent among physicians compared with non-medical participants (*p* < 0.05; Fig 1B). Among physicians, 42% (n = 163) were single, 32% (n = 125) were married, 2% (n = 8) were widowed, and 24% (n = 94) were divorced. In contrast, the non-medical group included 35% (n = 84) single, 55.8% (n = 134) married, 1.7% (n = 4) widowed, and 7.5% (n = 18) divorced participants (Fig 1B).

Gender distribution did not differ significantly between groups, with females comprising 58% of physicians and 61% of non-medical participants.

### Stress and depression prevalence

In unadjusted comparisons, physicians reported higher levels of stress and depressive symptoms than non-medical participants (*p* < 0.05). Stress was reported by 92% (n = 358) of physicians compared with 67% (n = 161) of non-medical participants.

**Fig 2:**
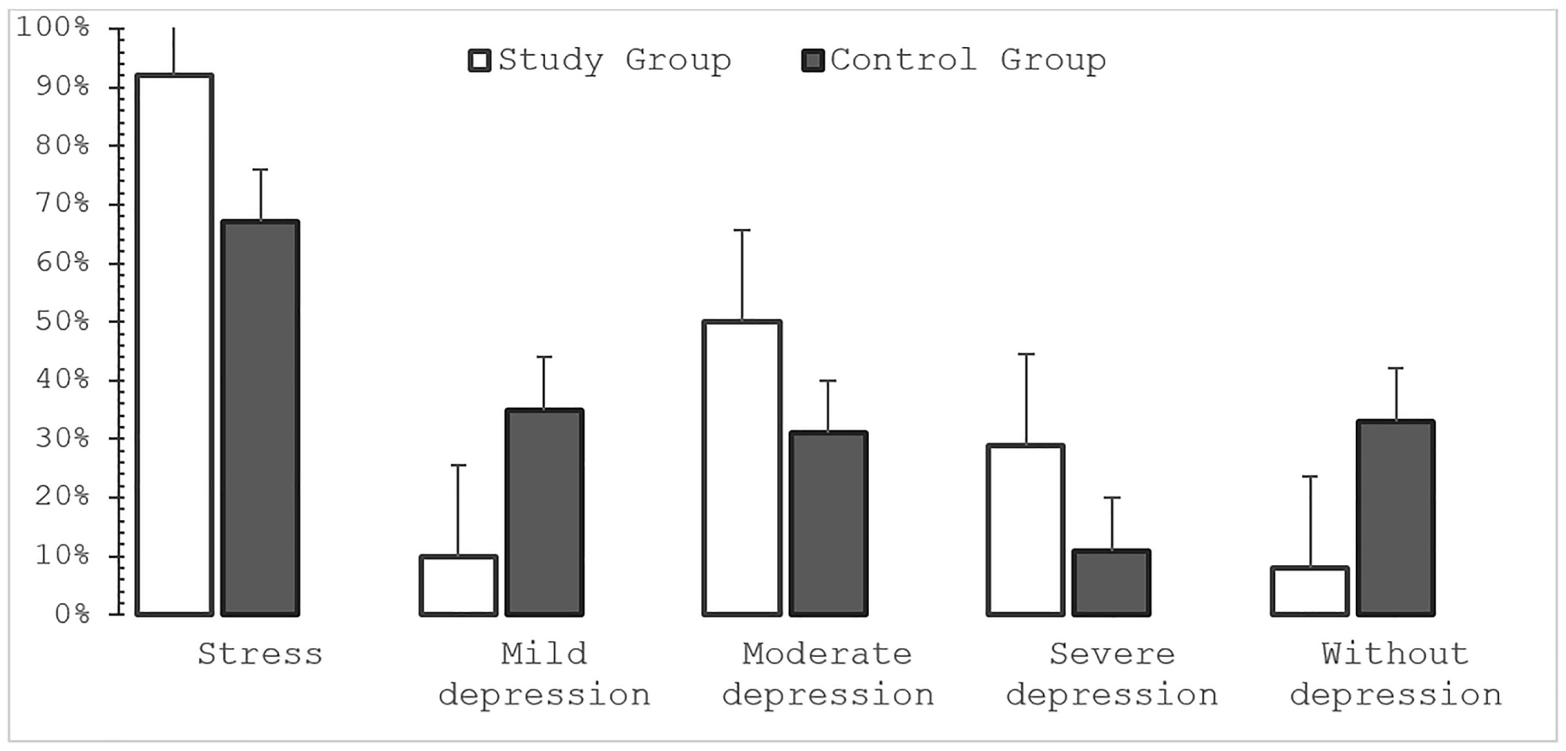
Stress and depression levels among medical and non-medical participants.

Moderate to severe depression was more prevalent among physicians (*p* < 0.05). Among physicians, 50% (n = 195) reported moderate depression and 29% (n = 113) severe depression. In the non-medical group, moderate and severe depression were reported by 31% (n = 74) and 11% (n = 27), respectively. In contrast, mild depression was more common among non-medical participants (35%, n = 84) than among physicians (10%, n = 39; *p* < 0.05) (Fig 2).

### Stress in relation to experience, gender, and marital status

Physicians were divided into three subgroups based on years of professional experience to examine associations between work tenure and stress levels. A significant negative correlation was observed between years of experience and stress prevalence (r = −0.7; *p* < 0.001). Stress prevalence was 75% among physicians with 0–10 years of experience, 55% among those with 10–20 years, and 48% among physicians with more than 20 years of experience.

Female physicians experienced higher stress compared with male physicians (OR = 1.68). In contrast, male physicians had higher odds of depression than female physicians (OR = 2.55). Married physicians reported higher stress prevalence than unmarried physicians (68% vs. 59%; *p* < 0.05). Age was also negatively correlated with stress levels (r = −0.5; *p* < 0.001).

### Stress and depression across medical specialties

**Fig 3:**
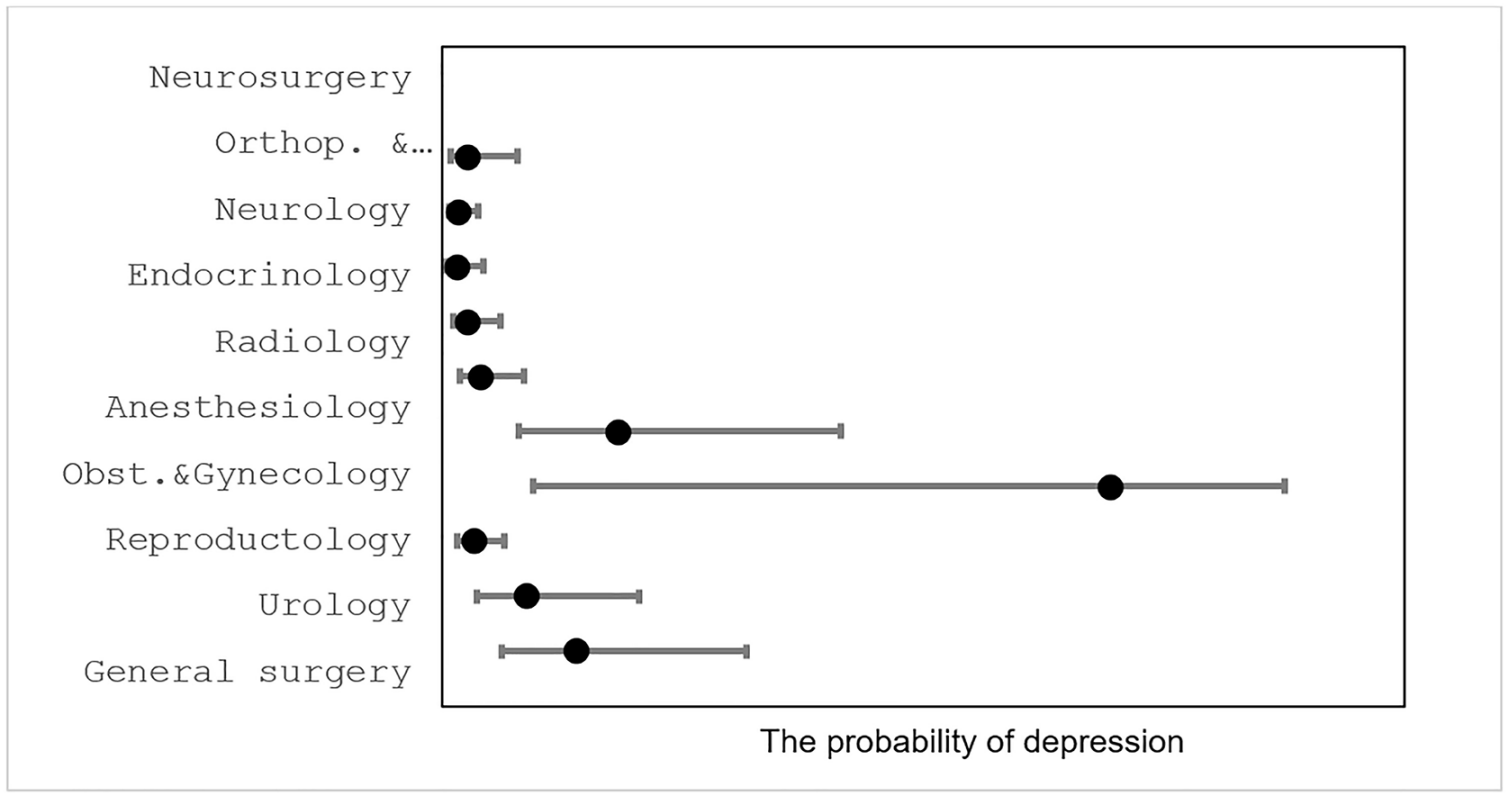
Odds ratios for depression across studied physician specialty groups.

In descriptive analyses, stress and depressive symptom burden varied across medical specialties. Among the specialties studied, obstetricians–gynecologists showed comparatively higher odds of depression in the unadjusted analyses (Fig 3). Elevated odds were also observed among anesthesiologists, general surgeons, and urologists, while physicians from the remaining specialties demonstrated lower odds of depression.

**Fig 4:**
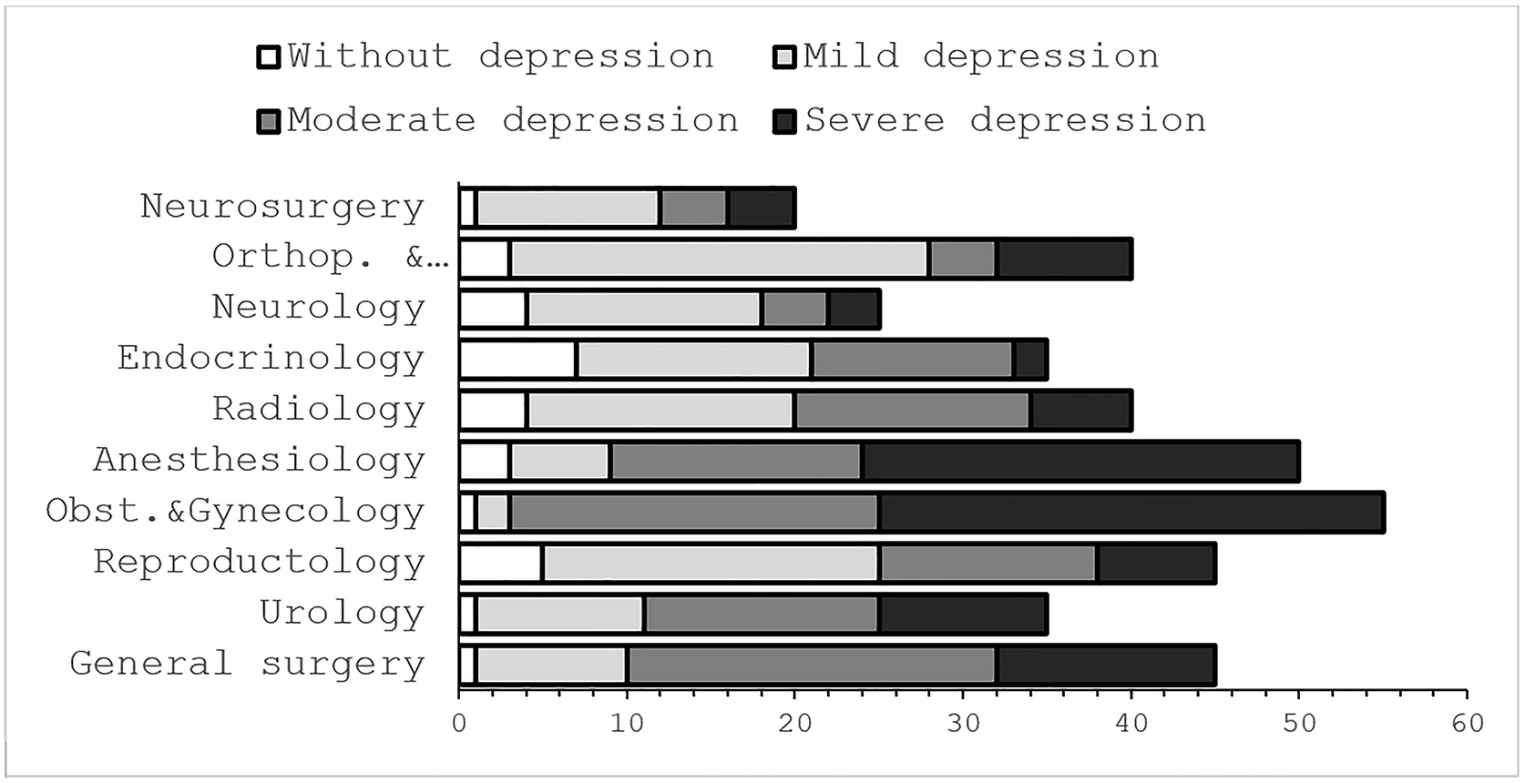
Distribution of PHQ-9 depression severity across medical specialties.

In the unadjusted analyses, stress levels varied by years of experience and gender among physicians. Comparative analysis further showed higher prevalence of stress and depression among obstetricians–gynecologists, general surgeons, urologists, and anesthesiologists compared with other medical specialties (*p* < 0.05). No significant differences in stress or depression prevalence were observed within this group of four specialties (Fig 4).

Severe depression prevalence was higher among obstetricians–gynecologists, urologists, and anesthesiologists compared with other specialties (*p* < 0.05), with no significant differences between these groups. Moderate depression prevalence was similar between obstetricians–gynecologists and general surgeons and higher than in other fields. Mild depression was more frequent among traumatologists, neurosurgeons, and neurologists. Endocrinologists and neurologists demonstrated lower depression prevalence compared with other specialties (*p* < 0.05) (Fig 4).

### Work-related factors and sleep disturbances

Correlation analysis showed significant associations between stress, depression, and work quality indicators. Physicians reported higher rates of stress-related work performance difficulties and interpersonal challenges compared with non-medical participants. These outcomes were reported more frequently among obstetricians–gynecologists, general surgeons, urologists, and anesthesiologists (*p* < 0.05).

Sleep disturbances were more common among physicians than non-medical participants and were most prevalent among obstetricians–gynecologists, general surgeons, and anesthesiologists (*p* < 0.05). Significant positive correlations were observed between stress, depression, and sleep disturbance.

### Workplace and personal stressors

**Fig 5:**
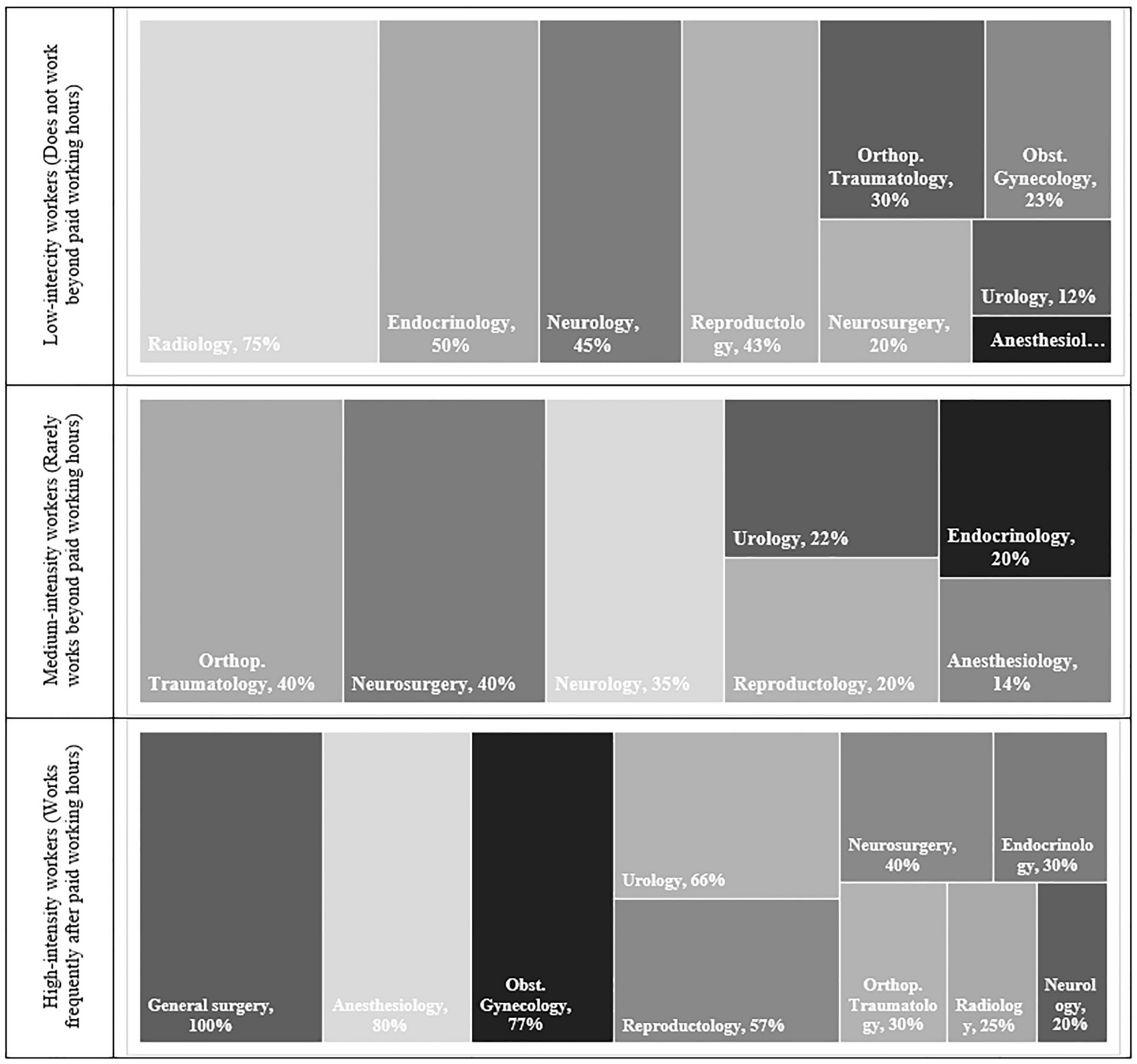
Prevalence of abnormal working hours and excessive workload across medical specialties.

Two main categories of stressors were identified among physicians: work-related stressors and personal life stressors. Work-related stressors included unsatisfactory work environments (80%), abnormal working hours and excessive workload (80%), and limited opportunities for professional development, financial support, and family time (60%). Stress related to interactions with work team members was also reported. Abnormal working hours and excessive workload were reported across all medical specialties (Fig 5).

Personal life stressors primarily involved difficulties maintaining work–life balance and perceived discrepancies between work effort and financial compensation.

**Fig 6:**
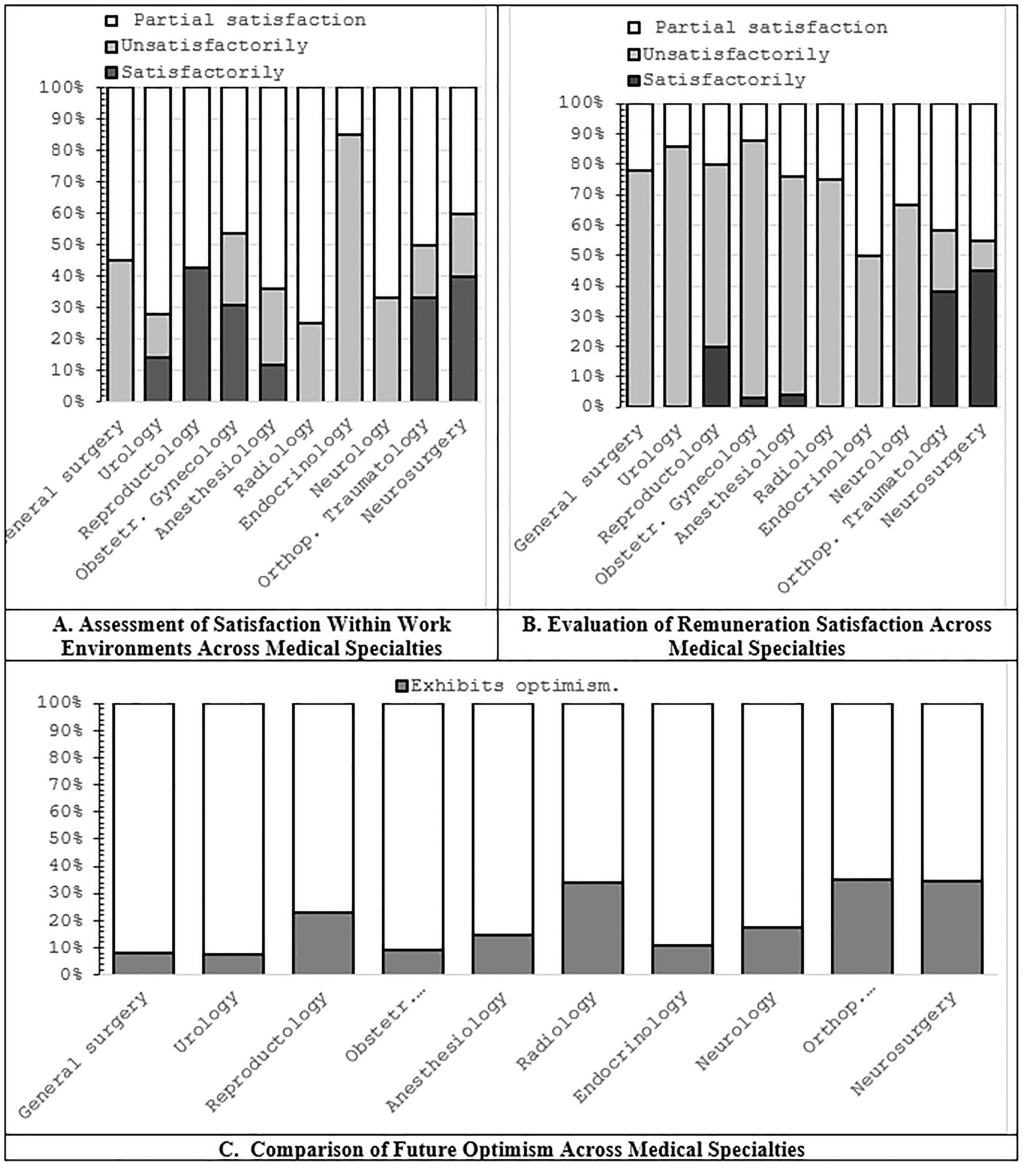
Work environment satisfaction, remuneration satisfaction, and future-oriented optimism across medical specialties.

Satisfaction with the work environment varied across specialties and was reported as a stress-related factor among all physician groups (Fig 6A). Remuneration satisfaction also differed by specialty, with orthopedic-traumatologists and neurosurgeons reporting higher satisfaction compared with other specialties (*p* < 0.05) (Fig 6B). Optimism regarding future professional expectations varied across specialties, with neurosurgeons, orthopedic-traumatologists, and radiologists reporting higher levels of optimism (Fig 6C).

Correlation analysis showed significant associations between remuneration satisfaction and stress levels (*p* < 0.05), as well as between optimism and happiness (*p* < 0.05). Age was negatively correlated with both optimism and happiness (*p* < 0.05).

## Discussion

The medical profession is inherently associated with high levels of occupational stress due to both the demanding nature of the work and specific personality traits common among physicians [1–6]. Literature consistently highlights that physicians, driven by high intelligence, empathy, and conscientiousness, may exhibit dysfunctional perfectionism, leading to overcommitment and difficulty disengaging from professional responsibilities. These characteristics may predispose physicians to emotional, cognitive, and physical exhaustion, as well as increased vulnerability to mental health challenges [1–5,7–21].

Consistent with these observations, the present study found higher reported levels of stress and depressive symptoms among physicians than among non-medical professionals in unadjusted comparisons. This finding is consistent with international research indicating that physicians are exposed to greater occupational stressors due to the complexity of their roles, sustained responsibility, and pressure to deliver high-quality care, often in resource-constrained environments. Meta-analyses report burnout prevalence exceeding 45% among physicians globally, with excessive workload and poor work–life balance frequently identified as major contributing factors [4,7,20,21].

Analyses in the study showed a positive association between stress and depressive symptoms, which should be interpreted as non-causal, and this pattern was also associated with poorer work-related functioning. The study may suggest that physicians in Georgia could experience different patterns of stress and depressive symptom burden across specialties. Physicians in anesthesiology, general surgery, obstetrics–gynecology, and urology showed higher levels of stress and depressive symptoms than other specialties in the unadjusted analyses, with obstetricians–gynecologists showing the highest odds of depression.

These findings may point to an important and underexplored aspect of physician mental health that warrants further investigation. Previous studies similarly report high psychological burden in obstetrics–gynecology due to emotional strain, long working hours, and the high stakes associated with maternal and neonatal outcomes [22,23]. Anesthesiologists and surgeons are frequently exposed to sustained high-intensity clinical environments requiring constant vigilance and rapid decision-making, which may contribute to emotional exhaustion and depressive symptoms [7,24]. Urologists often manage sensitive and long-term clinical conditions, which may further increase emotional burden [25].

Physicians in these high-demand specialties also reported lower levels of optimism and happiness compared with other fields. In contrast, neurosurgeons, orthopedic-traumatologists, and radiologists demonstrated higher levels of optimism. These differences may reflect variations in workload structure, career stability, perceived professional autonomy, and access to institutional resources across specialties. Such findings may underscore the role of job characteristics and organizational factors in shaping physicians’ psychological well-being, although causal interpretation is limited by the study design.

Interestingly, an inverse association was observed between years of professional experience and stress levels, which may suggest that more experienced physicians could develop adaptive coping strategies, clinical confidence, and resilience over time. Younger physicians reported higher stress levels and lower optimism, potentially reflecting early-career pressures, financial insecurity, and limited professional autonomy. These challenges may contribute to reduced career satisfaction and increased risk of workforce attrition, including migration or departure from clinical practice, particularly in high-demand specialties. Similar findings have been reported in previous studies, although some investigations report contrasting results [18,20,21,26].

These patterns may have potential implications for healthcare workforce sustainability, particularly in settings facing demographic challenges and increasing healthcare demands. Previous research suggests that familiarity with clinical environments and enhanced problem-solving abilities may contribute to greater resilience among more experienced physicians [20,21]. However, some studies report contradictory findings [18,26].

Work-related and personal life stressors emerged in the study as important contributors to physician distress. Work-related stressors were highly prevalent and included unsatisfactory work environments, abnormal working hours, excessive workload, and limited opportunities for professional development and family time. Dissatisfaction with remuneration was also associated with higher stress levels. In the Georgian healthcare context, relatively low compensation may require physicians to work across multiple healthcare institutions to maintain adequate income, resulting in extended working hours and reduced opportunities for rest and recovery. These structural and economic factors may be associated with greater occupational stress.

Gender- and marital-status–related differences were also observed. Female physicians reported higher stress levels, whereas male physicians demonstrated higher odds of depression. These findings are consistent with some studies suggesting that women in medicine may experience greater role strain due to combined professional and family responsibilities, while men may be less likely to seek psychological support, potentially contributing to higher depression prevalence [18,21,26–28]. However, findings in the literature are mixed, indicating that gender-related mental health outcomes among physicians are influenced by multiple contextual factors [18,21,26–28].

The study found that married physicians reported higher stress levels than unmarried colleagues, possibly reflecting challenges in balancing professional demands with family responsibilities. However, evidence regarding marital status and physician stress remains inconsistent, with some studies suggesting that institutional factors and workload may play a more prominent role than marital status alone [27].

The study also identified associations between stress, depression, and sleep disturbances. Higher stress levels were associated with poorer sleep quality, reduced happiness, and lower optimism. However, sleep disturbances may reflect broader occupational stressors rather than acting as an isolated cause of psychological distress. Prior research suggests that interventions focusing solely on sleep improvement may be insufficient unless accompanied by organizational changes addressing workload, working hours, and institutional support [26–30].

Similar to other studies, the present study found that dissatisfaction with remuneration was associated with higher stress levels among physicians. While salary alone may not determine professional productivity, adequate and fair compensation is important for meeting personal and family needs and may contribute to reduced occupational stress. These findings may suggest the relevance of remuneration as a structural factor related to physician well-being.

This study provides important context-specific evidence on physician mental health in Georgia. The findings may suggest the relevance of targeted, specialty-sensitive approaches addressing workload management, remuneration, institutional support, and early-career stressors. Future research using longitudinal designs and validated measurement frameworks would help further evaluate these associations.

### Limitations

Several limitations should be considered when interpreting these findings. The cross-sectional design precludes causal inference, and the use of non-probability convenience sampling may introduce selection bias and limit generalizability. Both physician and non-medical samples were recruited primarily from Tbilisi and are not nationally representative. Mental health outcomes and work-related factors were assessed using self-reported measures, which may be subject to reporting bias.

In addition, no confirmatory or exploratory factor analysis was conducted to formally evaluate the factor structure of the translated instruments; therefore, results should be interpreted as screening-based rather than diagnostic. The study also involved a large number of statistical comparisons across relatively small subgroups, increasing the risk of chance findings, particularly for specialty-specific differences. Finally, the absence of a validated multidimensional quality-of-life instrument (e.g., WHOQOL-BREF or SF-36) represents a limitation; accordingly, the findings should be interpreted as reflecting stress, depressive symptoms, and selected work-related and psychosocial well-being indicators rather than a comprehensive assessment of quality of life.

Because this study was cross-sectional, convenience-sampled, and based on unadjusted analyses, the findings should be interpreted as descriptive and hypothesis-generating rather than explanatory.

## Conclusion

Physicians in this study reported significantly higher levels of stress and depressive symptoms compared with the non-medical comparison group, with potential implications for occupational well-being, work-related functioning, and future professional outlook. These findings may suggest the relevance of addressing physician mental health as a component of healthcare system sustainability in Georgia.

The results may support further consideration of strategies aimed at supporting physician well-being, including organizational approaches that address workload, working hours, and access to psychosocial support. Efforts to strengthen professional development opportunities and supportive work environments may also be relevant. Addressing these factors may contribute to supporting a resilient healthcare workforce and the long-term functioning of the healthcare system in Georgia. Future longitudinal and multivariable studies are needed to better evaluate these relationships.

## Supporting information

S1 Database Appendix of the article. (DOCX)

S2 Database Minimal_dataset. (XLSX)
