# Supplementary material for: Work-life realities of doctors in Georgia and their impact on mental health and work-related well-being: A cross-sectional study
Source: PLoS One. 2026 May 29;21(5):e0349641. doi: 10.1371/journal.pone.0349641 (PMC13220998; doi:10.1371/journal.pone.0349641)
Supplement: S1 Database — (DOCX) [file pone.0349641.s001.docx]

**Supporting Information Caption**

**S1 Appendix. Database of the article “Work-Life Realities of Doctors in Georgia and Their Impact on Mental Health and Work-Related Well-Being: A Cross-Sectional Study”. The supporting file contains anonymized appendix data on participant structural characteristics (age, marital status, and gender) and the distribution of stress and depression levels among physician participants and non-physician comparison participants.**

**Appendix of the article: “Work-Life Realities of Doctors in Georgia and Their Impact on Mental Health and Work-Related Well-Being: A Cross-Sectional Study”**

The database provided in the appendix contains information on structural characteristics and the distribution of stress and depression levels among participant groups, including physicians and non-physicians (the comparison group).

1) Structure: (Age, Marital Status and Gender) of the Physician participants or the “study group”. Abbreviations: M - Married; S - Single, D - Divorced, W - Widowed.

| Age group: 25-30 years | | | Age group: 30-35 years | | | Age group: 35-60 years | | | Age group: Retirement age | | |
| --- | --- | --- | --- | --- | --- | --- | --- | --- | --- | --- | --- |
| Age | Marital Status | Gender | Age | Marital Status | Gender | Age | Marital Status | Gender | Age | Marital Status | Gender |
| 25 | M | Male | 34 | D | Female | 51 | M | Female | 64 | M | Female |
| 28 | M | Female | 31 | D | Female | 48 | M | Female | 65 | M | Male |
| 28 | M | Male | 31 | S | Male | 42 | S | Male | 71 | M | Female |
| 28 | D | Female | 33 | D | Female | 40 | D | Female | 69 | S | Female |
| 27 | M | Male | 33 | S | Female | 49 | D | Male | 68 | M | Female |
| 27 | S | Female | 35 | M | Male | 45 | M | Female | 70 | S | Female |
| 26 | S | Female | 31 | S | Female | 48 | D | Male | 61 | S | Male |
| 25 | S | Female | 30 | M | Male | 59 | M | Female | 64 | M | Female |
| 28 | M | Female | 31 | S | Female | 41 | S | Male | 63 | D | Female |
| 29 | S | Male | 33 | S | Male | 36 | S | Female | 68 | S | Male |
| 29 | S | Male | 34 | S | Female | 51 | D | Female | 62 | D | Female |
| 28 | S | Female | 35 | S | Female | 52 | S | Female | 74 | S | Female |
| 29 | M | Male | 30 | M | Male | 55 | M | Female | 72 | W | Female |
| 27 | S | Female | 32 | S | Male | 37 | M | Female | 74 | M | Male |
| 26 | S | Female | 34 | M | Female | 60 | S | Female | 63 | S | Male |
| 27 | S | Male | 35 | M | Male | 38 | D | Female | 70 | S | Female |
| 29 | S | Female | 30 | M | Female | 52 | D | Male | 70 | M | Female |
| 26 | S | Female | 35 | D | Male | 49 | D | Male | 65 | M | Female |
| 26 | S | Female | 32 | D | Male | 48 | S | Female | 72 | M | Male |
| 29 | S | Male | 31 | S | Male | 42 | S | Female | 63 | D | Female |
| 25 | M | Male | 32 | S | Female | 44 | M | Male | Summary | | |
| 25 | S | Female | 34 | S | Male | 47 | S | Female | Character groups | | Share (%) |
| 28 | S | Male | 33 | D | Female | 43 | M | Male | Female | | 58 |
| 28 | S | Female | 34 | S | Female | 57 | S | Female | Male | | 42 |
| 25 | S | Female | 33 | M | Male | 48 | S | Male | Married | | 32 |
| 28 | S | Female | 35 | M | Male | 45 | D | Female | Single | | 32 |
| 28 | S | Female | 34 | D | Male | 54 | W | Female | Divorced | | 34 |
| 27 | S | Male | 33 | D | Male | 58 | D | Female | Widowed | | 2 |
| 29 | S | Female | 32 | S | Female | 48 | S | Male | Age group: 25-30 years | | 18 |
| 27 | S | Male | 30 | D | Male | 60 | M | Female | Age group: 30-35 years | | 37 |
| 26 | S | Female | 33 | S | Female | 39 | M | Female | Age group: 35-60 years | | 40 |
| 27 | S | Male | 33 | S | Female | 49 | S | Male | Age group: Retirement age | | 5 |
| 26 | M | Male | 32 | D | Female | 38 | D | Male |  |  |  |
| 28 | M | Female | 32 | D | Female | 42 | S | Female |  |  |  |
| 28 | S | Male | 31 | S | Male | 48 | S | Female |  |  |  |
| 29 | S | Male | 32 | S | Male | 39 | D | Female |  |  |  |
| 26 | S | Male | 30 | S | Female | 49 | M | Female |  |  |  |
| 26 | S | Male | 32 | D | Male | 45 | M | Male |  |  |  |
| 29 | S | Female | 31 | S | Male | 51 | M | Female |  |  |  |
| 26 | S | Male | 34 | S | Female | 52 | M | Female |  |  |  |
| 26 | S | Male | 32 | M | Female | 44 | M | Female |  |  |  |
| 25 | M | Female | 31 | M | Male | 42 | S | Female |  |  |  |
| 27 | S | Female | 30 | D | Female | 60 | M | Female |  |  |  |
| 29 | M | Male | 34 | S | Male | 43 | M | Male |  |  |  |
| 28 | S | Female | 31 | M | Male | 54 | M | Female |  |  |  |
| 26 | S | Female | 32 | D | Female | 60 | S | Male |  |  |  |
| 28 | D | Female | 33 | D | Female | 39 | M | Female |  |  |  |
| 29 | S | Male | 35 | M | Female | 59 | D | Male |  |  |  |
| 28 | S | Male | 32 | S | Female | 37 | M | Female |  |  |  |
| 29 | S | Female | 31 | M | Male | 41 | S | Male |  |  |  |
| 29 | S | Male | 34 | M | Female | 59 | D | Female |  |  |  |
| 28 | S | Female | 34 | D | Male | 59 | D | Female |  |  |  |
| 29 | S | Male | 32 | M | Female | 55 | D | Female |  |  |  |
| 26 | M | Male | 35 | S | Female | 45 | W | Female |  |  |  |
| 25 | M | Male | 30 | M | Male | 45 | S | Female |  |  |  |
| 28 | S | Female | 32 | W | Female | 36 | M | Female |  |  |  |
| 25 | M | Male | 32 | S | Male | 48 | M | Female |  |  |  |
| 28 | M | Male | 31 | S | Female | 58 | M | Female |  |  |  |
| 25 | S | Male | 35 | D | Male | 42 | S | Female |  |  |  |
| 29 | M | Male | 30 | M | Male | 46 | D | Female |  |  |  |
| 25 | S | Female | 35 | M | Female | 44 | D | Male |  |  |  |
| 25 | M | Male | 34 | D | Female | 56 | S | Female |  |  |  |
| 26 | S | Male | 33 | M | Male | 50 | M | Male |  |  |  |
| 27 | S | Male | 35 | M | Female | 52 | S | Female |  |  |  |
| 29 | M | Female | 32 | S | Male | 57 | M | Male |  |  |  |
| 28 | S | Female | 33 | D | Female | 58 | M | Female |  |  |  |
| 25 | S | Female | 30 | S | Female | 42 | M | Male |  |  |  |
| 27 | S | Male | 33 | M | Female | 40 | M | Female |  |  |  |
| 27 | M | Female | 31 | M | Female | 43 | S | Female |  |  |  |
| 25 | M | Male | 31 | S | Female | 39 | D | Female |  |  |  |
|  |  |  | 35 | D | Female | 44 | S | Female |  |  |  |
|  |  |  | 33 | M | Male | 45 | S | Female |  |  |  |
|  |  |  | 32 | D | Male | 41 | M | Female |  |  |  |
|  |  |  | 32 | D | Female | 51 | D | Male |  |  |  |
|  |  |  | 33 | M | Female | 52 | D | Male |  |  |  |
|  |  |  | 30 | S | Female | 37 | M | Female |  |  |  |
|  |  |  | 33 | S | Male | 40 | S | Female |  |  |  |
|  |  |  | 30 | S | Male | 41 | S | Female |  |  |  |
|  |  |  | 35 | S | Male | 38 | M | Male |  |  |  |
|  |  |  | 31 | S | Female | 58 | M | Female |  |  |  |
|  |  |  | 35 | D | Female | 46 | D | Male |  |  |  |
|  |  |  | 31 | M | Male | 44 | M | Female |  |  |  |
|  |  |  | 34 | S | Female | 41 | S | Male |  |  |  |
|  |  |  | 35 | M | Male | 42 | S | Female |  |  |  |
|  |  |  | 33 | S | Male | 38 | D | Male |  |  |  |
|  |  |  | 33 | S | Male | 40 | S | Female |  |  |  |
|  |  |  | 31 | D | Female | 40 | D | Male |  |  |  |
|  |  |  | 32 | D | Male | 41 | S | Female |  |  |  |
|  |  |  | 31 | M | Female | 50 | S | Male |  |  |  |
|  |  |  | 33 | M | Female | 58 | M | Female |  |  |  |
|  |  |  | 31 | D | Male | 51 | S | Male |  |  |  |
|  |  |  | 31 | M | Male | 40 | D | Female |  |  |  |
|  |  |  | 31 | M | Female | 38 | D | Male |  |  |  |
|  |  |  | 34 | S | Female | 60 | S | Female |  |  |  |
|  |  |  | 30 | M | Female | 38 | S | Female |  |  |  |
|  |  |  | 30 | S | Female | 56 | D | Female |  |  |  |
|  |  |  | 33 | S | Male | 41 | S | Male |  |  |  |
|  |  |  | 35 | S | Female | 55 | D | Female |  |  |  |
|  |  |  | 32 | S | Male | 50 | S | Male |  |  |  |
|  |  |  | 30 | S | Male | 40 | S | Male |  |  |  |
|  |  |  | 32 | D | Male | 57 | S | Female |  |  |  |
|  |  |  | 35 | D | Male | 50 | S | Female |  |  |  |
|  |  |  | 30 | D | Male | 42 | M | Female |  |  |  |
|  |  |  | 31 | S | Female | 48 | D | Male |  |  |  |
|  |  |  | 32 | M | Male | 59 | D | Female |  |  |  |
|  |  |  | 32 | D | Female | 40 | S | Female |  |  |  |
|  |  |  | 35 | S | Female | 59 | M | Male |  |  |  |
|  |  |  | 33 | D | Female | 37 | D | Male |  |  |  |
|  |  |  | 32 | D | Male | 37 | S | Female |  |  |  |
|  |  |  | 31 | D | Female | 51 | M | Female |  |  |  |
|  |  |  | 34 | D | Female | 55 | M | Female |  |  |  |
|  |  |  | 30 | D | Male | 60 | S | Female |  |  |  |
|  |  |  | 33 | D | Male | 37 | S | Female |  |  |  |
|  |  |  | 31 | D | Male | 45 | D | Male |  |  |  |
|  |  |  | 33 | S | Male | 43 | D | Male |  |  |  |
|  |  |  | 31 | M | Male | 49 | M | Female |  |  |  |
|  |  |  | 32 | D | Female | 53 | D | Female |  |  |  |
|  |  |  | 33 | S | Female | 57 | S | Female |  |  |  |
|  |  |  | 34 | D | Female | 38 | D | Female |  |  |  |
|  |  |  | 35 | S | Male | 43 | M | Male |  |  |  |
|  |  |  | 33 | S | Female | 38 | S | Male |  |  |  |
|  |  |  | 33 | S | Male | 55 | S | Female |  |  |  |
|  |  |  | 34 | S | Female | 46 | M | Female |  |  |  |
|  |  |  | 30 | M | Female | 39 | M | Female |  |  |  |
|  |  |  | 33 | D | Female | 47 | M | Female |  |  |  |
|  |  |  | 35 | D | Male | 54 | M | Female |  |  |  |
|  |  |  | 31 | S | Female | 55 | D | Male |  |  |  |
|  |  |  | 30 | D | Female | 38 | D | Male |  |  |  |
|  |  |  | 33 | D | Male | 58 | D | Female |  |  |  |
|  |  |  | 31 | M | Female | 53 | S | Female |  |  |  |
|  |  |  | 31 | M | Male | 50 | M | Female |  |  |  |
|  |  |  | 32 | D | Male | 57 | D | Male |  |  |  |
|  |  |  | 32 | M | Female | 59 | D | Male |  |  |  |
|  |  |  | 33 | D | Male | 58 | D | Female |  |  |  |
|  |  |  | 34 | M | Male | 51 | S | Female |  |  |  |
|  |  |  | 35 | S | Male | 46 | S | Female |  |  |  |
|  |  |  | 34 | D | Male | 37 | S | Female |  |  |  |
|  |  |  | 30 | S | Female | 36 | D | Male |  |  |  |
|  |  |  | 32 | M | Male | 46 | S | Female |  |  |  |
|  |  |  | 30 | D | Female | 42 | S | Male |  |  |  |
|  |  |  | 32 | M | Male | 55 | S | Female |  |  |  |
|  |  |  | 30 | S | Female | 44 | M | Male |  |  |  |
|  |  |  | 32 | M | Female | 49 | D | Female |  |  |  |
|  |  |  | 34 | M | Male | 51 | D | Male |  |  |  |
|  |  |  | 32 | D | Female | 48 | M | Female |  |  |  |
|  |  |  |  |  |  | 43 | M | Female |  |  |  |
|  |  |  |  |  |  | 56 | S | Female |  |  |  |
|  |  |  |  |  |  | 56 | M | Female |  |  |  |
|  |  |  |  |  |  | 38 | M | Female |  |  |  |
|  |  |  |  |  |  | 48 | M | Male |  |  |  |
|  |  |  |  |  |  | 47 | D | Female |  |  |  |
|  |  |  |  |  |  | 39 | M | Female |  |  |  |
|  |  |  |  |  |  | 53 | M | Male |  |  |  |
|  |  |  |  |  |  | 49 | M | Female |  |  |  |
|  |  |  |  |  |  | 42 | S | Female |  |  |  |

2) Structure: (Age, Marital Status and Gender) of the Non-Physician participants or the “comparison group”. Abbreviations: M - Married; S - Single, D - Divorced, W - Widowed.

| Age group: 25-30 years | | | Age group: 30-35 years | | | Age group: 35-60 years | | | Age group: Retirement age | | |
| --- | --- | --- | --- | --- | --- | --- | --- | --- | --- | --- | --- |
| Age | Marital Status | Gender | Age | Marital Status | Gender | Age | Marital Status | Gender | Age | Marital Status | Gender |
| 27 | S | Male | 33 | S | Male | 42 | M | Female | 61 | W | Female |
| 28 | S | Female | 34 | S | Male | 41 | M | Female | 63 | M | Male |
| 25 | S | Male | 32 | M | Female | 38 | D | Female | 67 | M | Female |
| 30 | M | Female | 31 | S | Male | 52 | M | Female | 62 | D | Female |
| 29 | M | Female | 35 | D | Male | 41 | M | Female | 62 | M | Male |
| 25 | S | Male | 33 | D | Male | 40 | M | Female | 65 | W | Female |
| 26 | S | Male | 33 | S | Male | 50 | M | Female | 66 | M | Female |
| 29 | M | Female | 35 | M | Female | 55 | M | Female | 64 | M | Female |
| 27 | D | Female | 34 | M | Female | 60 | W | Female | Summary | | |
| 30 | S | Male | 35 | M | Female | 55 | M | Female | Character groups | Share (%) | |
| 25 | S | Male | 31 | D | Female | 42 | M | Female | Female | 61 | |
| 30 | M | Female | 33 | M | Female | 36 | S | Male | Male | 39 | |
| 26 | S | Female | 32 | M | Female | 55 | M | Male | Married | 55.8 | |
| 29 | S | Male | 34 | W | Female | 50 | M | Female | Single | 35 | |
| 27 | S | Male | 35 | M | Female | 49 | M | Female | Divorced | 7.5 | |
| 28 | M | Female | 33 | S | Male | 42 | D | Male | Widowed | 1.7 | |
| 27 | S | Female | 31 | S | Male | 50 | M | Female | Age group: 25-30 years | 31 | |
| 30 | M | Female | 32 | S | Female | 36 | S | Female | Age group: 30-35 years | 32 | |
| 30 | M | Female | 33 | M | Female | 39 | M | Female | Age group: 35-60 years | 34 | |
| 29 | M | Male | 34 | D | Female | 36 | M | Female | Age group: Retirement age | 3 | |
| 30 | M | Female | 35 | M | Female | 42 | M | Male |  |  |  |
| 25 | S | Male | 34 | M | Female | 54 | M | Female |  |  |  |
| 27 | M | Male | 31 | S | Male | 37 | M | Female |  |  |  |
| 30 | S | Male | 31 | M | Female | 44 | S | Female |  |  |  |
| 28 | S | Male | 33 | M | Female | 37 | S | Male |  |  |  |
| 26 | M | Male | 32 | M | Female | 41 | M | Female |  |  |  |
| 28 | S | Male | 33 | S | Female | 42 | M | Female |  |  |  |
| 30 | S | Male | 33 | M | Female | 42 | M | Female |  |  |  |
| 30 | S | Male | 33 | S | Male | 57 | M | Female |  |  |  |
| 30 | M | Female | 31 | S | Male | 39 | M | Female |  |  |  |
| 25 | S | Female | 31 | M | Male | 58 | M | Female |  |  |  |
| 25 | M | Female | 33 | M | Female | 55 | M | Male |  |  |  |
| 25 | S | Male | 34 | S | Female | 54 | M | Female |  |  |  |
| 29 | M | Female | 32 | M | Female | 50 | D | Male |  |  |  |
| 28 | S | Male | 32 | M | Female | 44 | M | Female |  |  |  |
| 27 | S | Male | 35 | M | Female | 44 | M | Female |  |  |  |
| 29 | M | Female | 31 | M | Female | 50 | M | Female |  |  |  |
| 25 | S | Male | 34 | M | Female | 55 | M | Female |  |  |  |
| 29 | S | Male | 34 | M | Female | 54 | D | Female |  |  |  |
| 29 | S | Female | 33 | S | Female | 57 | M | Female |  |  |  |
| 25 | S | Male | 32 | M | Female | 55 | M | Male |  |  |  |
| 26 | S | Female | 33 | S | Female | 54 | M | Female |  |  |  |
| 30 | S | Male | 32 | M | Female | 41 | M | Male |  |  |  |
| 29 | M | Female | 31 | S | Male | 56 | M | Female |  |  |  |
| 27 | S | Female | 31 | S | Male | 55 | M | Male |  |  |  |
| 29 | M | Female | 35 | M | Female | 51 | D | Female |  |  |  |
| 27 | S | Female | 31 | S | Male | 36 | M | Female |  |  |  |
| 28 | M | Female | 32 | M | Female | 43 | M | Female |  |  |  |
| 26 | S | Male | 35 | M | Female | 53 | M | Male |  |  |  |
| 30 | S | Male | 31 | M | Female | 50 | D | Male |  |  |  |
| 28 | S | Male | 32 | M | Female | 56 | M | Female |  |  |  |
| 28 | S | Male | 35 | M | Female | 38 | M | Female |  |  |  |
| 29 | M | Male | 35 | M | Female | 46 | M | Female |  |  |  |
| 30 | S | Female | 32 | D | Male | 49 | M | Female |  |  |  |
| 30 | M | Female | 35 | M | Female | 37 | S | Female |  |  |  |
| 27 | S | Male | 35 | M | Female | 49 | M | Male |  |  |  |
| 27 | S | Male | 34 | M | Female | 59 | M | Female |  |  |  |
| 29 | S | Male | 32 | S | Male | 54 | M | Female |  |  |  |
| 29 | S | Male | 35 | S | Male | 50 | M | Female |  |  |  |
| 25 | S | Male | 31 | M | Female | 45 | M | Male |  |  |  |
| 27 | S | Female | 35 | S | Male | 37 | M | Female |  |  |  |
| 30 | M | Female | 34 | S | Male | 40 | D | Female |  |  |  |
| 30 | M | Female | 33 | M | Female | 36 | S | Male |  |  |  |
| 27 | S | Male | 31 | S | Male | 41 | M | Female |  |  |  |
| 29 | S | Male | 35 | M | Male | 38 | M | Female |  |  |  |
| 29 | S | Male | 31 | S | Female | 59 | W | Female |  |  |  |
| 30 | S | Male | 32 | S | Male | 38 | S | Male |  |  |  |
| 25 | D | Female | 32 | S | Male | 45 | M | Female |  |  |  |
| 28 | S | Female | 34 | M | Female | 55 | M | Female |  |  |  |
| 28 | S | Male | 35 | M | Female | 57 | M | Female |  |  |  |
| 28 | M | Female | 33 | S | Male | 52 | M | Female |  |  |  |
| 27 | D | Female | 34 | M | Female | 41 | S | Male |  |  |  |
| 25 | S | Male | 32 | S | Male | 47 | M | Female |  |  |  |
| 29 | M | Female | 31 | S | Male | 58 | D | Female |  |  |  |
|  |  |  | 34 | M | Female | 49 | M | Female |  |  |  |
|  |  |  | 34 | S | Female | 45 | M | Female |  |  |  |
|  |  |  | 35 | M | Female | 42 | M | Female |  |  |  |
|  |  |  |  |  |  | 45 | M | Female |  |  |  |
|  |  |  |  |  |  | 46 | M | Female |  |  |  |
|  |  |  |  |  |  | 45 | M | Female |  |  |  |
|  |  |  |  |  |  | 44 | M | Female |  |  |  |

3) Distribution of the stress and depression levels within the group of the physician participants or the “Study group”

| **Participant** | **Stress status** | **Mild depression** | **Moderate depression** | **Severe depression** | **Participant** | **Stress status** | **Mild depression** | **Moderate depression** | **Severe depression** |
| --- | --- | --- | --- | --- | --- | --- | --- | --- | --- |
| **№1** | Yes | No | Yes | Yes | **№196** | Yes | No | No | No |
| **№2** | Yes | No | No | No | **№197** | Yes | No | No | No |
| **№3** | Yes | No | No | No | **№198** | Yes | No | No | No |
| **№4** | Yes | No | Yes | Yes | **№199** | Yes | No | Yes | No |
| **№5** | Yes | Yes | Yes | Yes | **№200** | Yes | No | Yes | No |
| **№6** | Yes | No | Yes | No | **№201** | Yes | No | Yes | Yes |
| **№7** | Yes | Yes | No | No | **№202** | Yes | No | Yes | No |
| **№8** | Yes | No | Yes | No | **№203** | Yes | No | No | Yes |
| **№9** | Yes | Yes | Yes | No | **№204** | Yes | No | No | Yes |
| **№10** | Yes | No | Yes | No | **№205** | Yes | No | No | Yes |
| **№11** | Yes | No | No | Yes | **№206** | Yes | No | No | No |
| **№12** | Yes | No | Yes | No | **№207** | Yes | No | Yes | Yes |
| **№13** | Yes | No | Yes | No | **№208** | Yes | No | Yes | No |
| **№14** | Yes | No | Yes | No | **№209** | No | No | No | No |
| **№15** | Yes | Yes | Yes | Yes | **№210** | Yes | No | No | No |
| **№16** | Yes | No | Yes | Yes | **№211** | Yes | No | No | No |
| **№17** | Yes | No | No | Yes | **№212** | Yes | No | No | No |
| **№18** | Yes | No | Yes | No | **№213** | Yes | No | No | No |
| **№19** | Yes | No | Yes | Yes | **№214** | Yes | No | Yes | Yes |
| **№20** | Yes | No | No | No | **№215** | Yes | No | Yes | Yes |
| **№21** | Yes | No | Yes | No | **№216** | Yes | No | Yes | Yes |
| **№22** | Yes | No | No | No | **№217** | No | No | No | No |
| **№23** | Yes | No | No | No | **№218** | Yes | No | Yes | No |
| **№24** | Yes | No | Yes | No | **№219** | Yes | Yes | No | Yes |
| **№25** | Yes | No | Yes | No | **№220** | Yes | No | Yes | Yes |
| **№26** | Yes | No | No | Yes | **№221** | Yes | No | Yes | No |
| **№27** | Yes | No | No | No | **№222** | Yes | No | Yes | No |
| **№28** | Yes | No | No | Yes | **№223** | Yes | No | Yes | Yes |
| **№29** | Yes | No | Yes | No | **№224** | Yes | No | No | Yes |
| **№30** | Yes | Yes | No | Yes | **№225** | Yes | No | No | No |
| **№31** | Yes | No | Yes | No | **№226** | Yes | No | Yes | No |
| **№32** | Yes | No | No | No | **№227** | Yes | No | Yes | No |
| **№33** | Yes | No | Yes | No | **№228** | Yes | No | Yes | No |
| **№34** | Yes | No | Yes | No | **№229** | Yes | No | Yes | Yes |
| **№35** | Yes | No | No | No | **№230** | Yes | No | No | No |
| **№36** | Yes | No | No | Yes | **№231** | Yes | No | No | No |
| **№37** | Yes | No | No | Yes | **№232** | Yes | No | Yes | Yes |
| **№38** | Yes | No | Yes | No | **№233** | Yes | No | Yes | No |
| **№39** | Yes | No | No | Yes | **№234** | Yes | No | No | Yes |
| **№40** | Yes | No | Yes | Yes | **№235** | Yes | No | No | Yes |
| **№41** | No | No | No | No | **№236** | Yes | No | No | No |
| **№42** | Yes | No | No | Yes | **№237** | Yes | No | No | No |
| **№43** | Yes | No | No | Yes | **№238** | Yes | No | No | No |
| **№44** | Yes | No | No | Yes | **№239** | Yes | No | Yes | Yes |
| **№45** | Yes | No | No | No | **№240** | Yes | No | No | No |
| **№46** | Yes | No | Yes | No | **№241** | Yes | No | No | No |
| **№47** | Yes | No | Yes | Yes | **№242** | Yes | No | Yes | No |
| **№48** | No | No | No | No | **№243** | Yes | No | No | No |
| **№49** | Yes | No | No | Yes | **№244** | Yes | No | No | No |
| **№50** | Yes | Yes | No | Yes | **№245** | Yes | No | Yes | No |
| **№51** | Yes | No | No | No | **№246** | Yes | Yes | No | No |
| **№52** | Yes | No | No | Yes | **№247** | Yes | No | No | No |
| **№53** | Yes | No | No | No | **№248** | Yes | No | No | No |
| **№54** | Yes | No | No | No | **№249** | Yes | No | Yes | Yes |
| **№55** | Yes | No | Yes | Yes | **№250** | Yes | No | Yes | No |
| **№56** | Yes | No | Yes | No | **№251** | Yes | No | No | No |
| **№57** | Yes | No | Yes | Yes | **№252** | Yes | Yes | No | No |
| **№58** | Yes | No | Yes | No | **№253** | Yes | No | Yes | No |
| **№59** | Yes | No | No | Yes | **№254** | Yes | No | No | No |
| **№60** | Yes | No | Yes | Yes | **№255** | Yes | No | No | No |
| **№61** | No | No | No | No | **№256** | Yes | No | No | No |
| **№62** | Yes | No | No | No | **№257** | Yes | No | Yes | No |
| **№63** | Yes | No | Yes | No | **№258** | Yes | No | Yes | Yes |
| **№64** | Yes | No | Yes | No | **№259** | Yes | No | Yes | No |
| **№65** | Yes | No | No | No | **№260** | No | No | No | No |
| **№66** | Yes | No | No | No | **№261** | Yes | No | No | Yes |
| **№67** | Yes | No | No | No | **№262** | No | No | No | No |
| **№68** | Yes | No | No | Yes | **№263** | Yes | Yes | Yes | Yes |
| **№69** | Yes | No | Yes | No | **№264** | Yes | No | Yes | No |
| **№70** | Yes | No | No | Yes | **№265** | Yes | No | Yes | No |
| **№71** | Yes | No | No | Yes | **№266** | Yes | No | No | Yes |
| **№72** | Yes | No | Yes | No | **№267** | Yes | No | Yes | No |
| **№73** | Yes | No | No | No | **№268** | Yes | No | Yes | Yes |
| **№74** | Yes | No | No | Yes | **№269** | Yes | No | Yes | No |
| **№75** | Yes | No | No | No | **№270** | Yes | No | Yes | No |
| **№76** | Yes | Yes | Yes | No | **№271** | Yes | No | Yes | Yes |
| **№77** | Yes | No | No | No | **№272** | Yes | No | Yes | No |
| **№78** | Yes | No | Yes | No | **№273** | Yes | No | No | No |
| **№79** | Yes | No | Yes | Yes | **№274** | Yes | Yes | Yes | No |
| **№80** | Yes | No | No | Yes | **№275** | Yes | No | No | No |
| **№81** | Yes | No | Yes | No | **№276** | Yes | No | No | No |
| **№82** | Yes | No | Yes | No | **№277** | Yes | No | No | Yes |
| **№83** | Yes | No | Yes | No | **№278** | Yes | No | Yes | No |
| **№84** | Yes | No | Yes | Yes | **№279** | Yes | No | Yes | Yes |
| **№85** | Yes | No | Yes | No | **№280** | Yes | No | No | Yes |
| **№86** | Yes | No | No | No | **№281** | Yes | No | Yes | No |
| **№87** | Yes | No | No | Yes | **№282** | Yes | No | No | No |
| **№88** | Yes | No | Yes | No | **№283** | Yes | Yes | Yes | No |
| **№89** | Yes | No | No | No | **№284** | Yes | No | No | No |
| **№90** | Yes | No | Yes | No | **№285** | Yes | No | No | No |
| **№91** | Yes | No | No | Yes | **№286** | Yes | No | Yes | Yes |
| **№92** | Yes | No | Yes | No | **№287** | Yes | No | Yes | No |
| **№93** | Yes | No | Yes | No | **№288** | Yes | No | No | No |
| **№94** | No | No | No | No | **№289** | Yes | No | No | Yes |
| **№95** | Yes | No | Yes | No | **№290** | No | No | No | No |
| **№96** | Yes | No | No | No | **№291** | Yes | No | Yes | No |
| **№97** | Yes | No | No | Yes | **№292** | Yes | No | Yes | Yes |
| **№98** | Yes | No | Yes | Yes | **№293** | Yes | Yes | No | Yes |
| **№99** | Yes | No | Yes | Yes | **№294** | Yes | No | No | Yes |
| **№100** | Yes | Yes | Yes | Yes | **№295** | Yes | No | Yes | No |
| **№101** | Yes | No | No | Yes | **№296** | Yes | No | No | Yes |
| **№102** | Yes | No | No | No | **№297** | Yes | No | Yes | No |
| **№103** | Yes | Yes | Yes | No | **№298** | No | No | No | No |
| **№104** | Yes | No | No | No | **№299** | Yes | Yes | No | No |
| **№105** | Yes | No | Yes | Yes | **№300** | Yes | No | Yes | No |
| **№106** | Yes | No | Yes | Yes | **№301** | Yes | No | Yes | No |
| **№107** | No | No | No | No | **№302** | Yes | No | No | No |
| **№108** | Yes | No | Yes | No | **№303** | Yes | Yes | Yes | Yes |
| **№109** | Yes | No | No | Yes | **№304** | Yes | No | No | Yes |
| **№110** | Yes | No | Yes | No | **№305** | Yes | No | No | No |
| **№111** | Yes | No | Yes | No | **№306** | Yes | No | Yes | Yes |
| **№112** | Yes | Yes | No | No | **№307** | Yes | No | Yes | No |
| **№113** | Yes | No | No | Yes | **№308** | Yes | No | Yes | No |
| **№114** | Yes | No | No | No | **№309** | No | No | No | No |
| **№115** | Yes | Yes | Yes | Yes | **№310** | Yes | Yes | Yes | Yes |
| **№116** | Yes | No | No | No | **№311** | Yes | No | Yes | No |
| **№117** | Yes | No | No | No | **№312** | Yes | No | Yes | No |
| **№118** | Yes | No | Yes | No | **№313** | Yes | No | No | No |
| **№119** | Yes | No | Yes | No | **№314** | Yes | No | Yes | No |
| **№120** | Yes | No | Yes | No | **№315** | No | No | No | No |
| **№121** | Yes | No | Yes | No | **№316** | Yes | Yes | Yes | No |
| **№122** | No | No | No | No | **№317** | Yes | Yes | Yes | No |
| **№123** | Yes | No | Yes | No | **№318** | Yes | No | Yes | No |
| **№124** | Yes | No | Yes | No | **№319** | Yes | No | No | No |
| **№125** | No | No | No | No | **№320** | Yes | No | No | No |
| **№126** | Yes | Yes | Yes | No | **№321** | Yes | No | No | No |
| **№127** | No | No | No | No | **№322** | Yes | No | No | No |
| **№128** | Yes | No | Yes | No | **№323** | Yes | No | No | Yes |
| **№129** | Yes | No | Yes | Yes | **№324** | No | No | No | No |
| **№130** | Yes | No | Yes | No | **№325** | Yes | No | Yes | No |
| **№131** | Yes | No | Yes | No | **№326** | Yes | Yes | Yes | No |
| **№132** | Yes | No | No | No | **№327** | Yes | No | No | No |
| **№133** | Yes | No | No | Yes | **№328** | Yes | No | Yes | No |
| **№134** | Yes | No | No | No | **№329** | Yes | No | No | No |
| **№135** | Yes | No | Yes | No | **№330** | Yes | No | Yes | No |
| **№136** | Yes | No | No | No | **№331** | Yes | No | Yes | No |
| **№137** | Yes | No | No | No | **№332** | Yes | No | Yes | No |
| **№138** | Yes | No | Yes | No | **№333** | Yes | No | No | Yes |
| **№139** | Yes | No | Yes | No | **№334** | Yes | Yes | No | No |
| **№140** | Yes | No | Yes | Yes | **№335** | Yes | No | No | No |
| **№141** | Yes | Yes | Yes | No | **№336** | Yes | No | No | No |
| **№142** | Yes | No | Yes | No | **№337** | Yes | No | No | No |
| **№143** | Yes | No | Yes | No | **№338** | Yes | No | Yes | No |
| **№144** | Yes | No | Yes | Yes | **№339** | Yes | No | Yes | No |
| **№145** | Yes | No | No | No | **№340** | Yes | No | Yes | No |
| **№146** | Yes | Yes | No | No | **№341** | Yes | No | Yes | No |
| **№147** | Yes | No | Yes | No | **№342** | Yes | Yes | Yes | No |
| **№148** | Yes | No | Yes | No | **№343** | Yes | No | No | No |
| **№149** | Yes | No | No | No | **№344** | Yes | No | No | No |
| **№150** | Yes | Yes | No | Yes | **№345** | Yes | No | Yes | Yes |
| **№151** | Yes | No | Yes | No | **№346** | No | No | No | No |
| **№152** | Yes | No | No | No | **№347** | Yes | No | No | No |
| **№153** | Yes | No | Yes | No | **№348** | Yes | Yes | No | No |
| **№154** | No | No | No | No | **№349** | Yes | No | Yes | No |
| **№155** | Yes | No | Yes | No | **№350** | Yes | No | No | Yes |
| **№156** | No | No | No | No | **№351** | Yes | No | Yes | No |
| **№157** | Yes | No | Yes | No | **№352** | Yes | No | Yes | No |
| **№158** | Yes | No | Yes | No | **№353** | Yes | Yes | No | No |
| **№159** | Yes | No | No | Yes | **№354** | No | No | No | No |
| **№160** | Yes | Yes | No | No | **№355** | Yes | No | Yes | No |
| **№161** | Yes | No | Yes | No | **№356** | No | No | No | No |
| **№162** | No | No | No | No | **№357** | Yes | No | No | No |
| **№163** | Yes | No | No | Yes | **№358** | Yes | No | Yes | Yes |
| **№164** | Yes | No | No | No | **№359** | No | No | No | No |
| **№165** | Yes | No | Yes | No | **№360** | Yes | No | Yes | Yes |
| **№166** | Yes | No | Yes | No | **№361** | Yes | No | Yes | Yes |
| **№167** | Yes | No | Yes | Yes | **№362** | Yes | No | Yes | No |
| **№168** | No | No | No | No | **№363** | Yes | No | No | No |
| **№169** | Yes | No | Yes | No | **№364** | Yes | No | Yes | Yes |
| **№170** | No | No | No | No | **№365** | No | No | No | No |
| **№171** | Yes | No | Yes | No | **№366** | Yes | No | Yes | No |
| **№172** | Yes | No | No | No | **№367** | Yes | No | Yes | Yes |
| **№173** | Yes | No | No | No | **№368** | Yes | No | Yes | No |
| **№174** | Yes | No | No | No | **№369** | Yes | No | Yes | No |
| **№175** | Yes | No | No | No | **№370** | Yes | No | Yes | No |
| **№176** | Yes | No | No | Yes | **№371** | Yes | No | Yes | No |
| **№177** | Yes | No | No | No | **№372** | No | No | No | Yes |
| **№178** | Yes | No | No | No | **№373** | Yes | No | Yes | Yes |
| **№179** | Yes | No | No | Yes | **№374** | No | No | No | No |
| **№180** | Yes | Yes | Yes | No | **№375** | Yes | No | No | No |
| **№181** | Yes | No | No | No | **№376** | Yes | No | Yes | Yes |
| **№182** | Yes | No | Yes | Yes | **№377** | Yes | No | Yes | No |
| **№183** | Yes | No | Yes | No | **№378** | Yes | No | No | No |
| **№184** | Yes | No | No | Yes | **№379** | Yes | No | No | No |
| **№185** | No | No | No | No | **№380** | Yes | No | Yes | No |
| **№186** | Yes | No | No | No | **№381** | Yes | No | No | No |
| **№187** | Yes | Yes | No | No | **№382** | Yes | No | No | No |
| **№188** | Yes | No | No | Yes | **№383** | Yes | No | No | No |
| **№189** | Yes | No | No | Yes | **№384** | Yes | Yes | Yes | No |
| **№190** | Yes | No | Yes | Yes | **№385** | Yes | No | Yes | No |
| **№191** | Yes | No | Yes | Yes | **№386** | No | No | No | No |
| **№192** | Yes | No | Yes | No | **№387** | Yes | Yes | Yes | Yes |
| **№193** | Yes | No | No | No | **№388** | Yes | Yes | Yes | Yes |
| **№194** | Yes | Yes | No | Yes | **№389** | Yes | No | Yes | No |
| **№195** | Yes | No | Yes | No | **№390** | Yes | No | No | No |

4) Distribution of the stress and depression levels within the group of the non-physician participants or the “Comparison group”

| **Participant** | **Stress status** | **Mild depression** | **Moderate depression** | **Severe depression** | **Participant** | **Stress status** | **Mild depression** | **Moderate depression** | **Severe depression** |
| --- | --- | --- | --- | --- | --- | --- | --- | --- | --- |
| **№1** | Yes | Yes | No | No | **№121** | Yes | No | Yes | No |
| **№2** | Yes | Yes | No | No | **№122** | No | No | No | No |
| **№3** | No | No | No | No | **№123** | Yes | No | No | No |
| **№4** | No | No | No | No | **№124** | No | No | No | No |
| **№5** | Yes | Yes | No | No | **№125** | Yes | No | No | No |
| **№6** | Yes | Yes | Yes | Yes | **№126** | No | No | No | No |
| **№7** | Yes | Yes | No | No | **№127** | No | No | No | No |
| **№8** | Yes | Yes | No | No | **№128** | No | No | No | No |
| **№9** | Yes | Yes | No | No | **№129** | Yes | No | Yes | No |
| **№10** | Yes | Yes | No | No | **№130** | No | No | No | No |
| **№11** | Yes | Yes | No | No | **№131** | Yes | Yes | No | No |
| **№12** | Yes | Yes | No | No | **№132** | Yes | No | No | No |
| **№13** | No | No | No | No | **№133** | No | No | No | No |
| **№14** | Yes | Yes | Yes | Yes | **№134** | Yes | No | Yes | No |
| **№15** | Yes | Yes | Yes | Yes | **№135** | Yes | No | Yes | No |
| **№16** | Yes | Yes | No | No | **№136** | No | No | No | No |
| **№17** | Yes | No | Yes | No | **№137** | No | No | No | No |
| **№18** | Yes | No | Yes | No | **№138** | No | No | No | No |
| **№19** | Yes | No | Yes | No | **№139** | Yes | No | Yes | No |
| **№20** | Yes | No | Yes | No | **№140** | Yes | No | Yes | No |
| **№21** | Yes | No | Yes | No | **№141** | Yes | No | Yes | Yes |
| **№22** | Yes | Yes | No | No | **№142** | No | No | No | No |
| **№23** | Yes | Yes | No | No | **№143** | Yes | Yes | No | No |
| **№24** | Yes | Yes | No | No | **№144** | Yes | No | No | No |
| **№25** | No | No | No | No | **№145** | Yes | Yes | No | No |
| **№26** | No | No | No | No | **№146** | No | No | No | No |
| **№27** | No | No | No | No | **№147** | No | No | No | No |
| **№28** | Yes | Yes | No | No | **№148** | No | No | No | No |
| **№29** | Yes | Yes | No | No | **№149** | Yes | No | Yes | Yes |
| **№30** | No | No | No | No | **№150** | No | No | No | No |
| **№31** | Yes | Yes | No | No | **№151** | Yes | No | No | No |
| **№32** | Yes | Yes | Yes | Yes | **№152** | Yes | No | No | No |
| **№33** | Yes | Yes | No | No | **№153** | Yes | No | No | No |
| **№34** | No | No | No | No | **№154** | No | No | No | No |
| **№35** | Yes | No | Yes | No | **№155** | Yes | No | Yes | Yes |
| **№36** | Yes | Yes | No | No | **№156** | Yes | No | Yes | No |
| **№37** | No | No | No | No | **№157** | Yes | Yes | Yes | No |
| **№38** | Yes | No | Yes | No | **№158** | Yes | Yes | No | No |
| **№39** | Yes | Yes | No | No | **№159** | No | No | No | No |
| **№40** | Yes | Yes | No | No | **№160** | Yes | No | Yes | No |
| **№41** | No | No | No | No | **№161** | No | No | No | No |
| **№42** | Yes | Yes | No | No | **№162** | Yes | Yes | No | No |
| **№43** | Yes | Yes | No | No | **№163** | Yes | Yes | No | No |
| **№44** | Yes | Yes | No | No | **№164** | No | No | No | No |
| **№45** | No | No | No | No | **№165** | No | No | No | No |
| **№46** | Yes | Yes | No | No | **№166** | Yes | Yes | No | No |
| **№47** | Yes | Yes | No | No | **№167** | Yes | Yes | No | No |
| **№48** | Yes | Yes | No | No | **№168** | Yes | No | Yes | Yes |
| **№49** | Yes | No | Yes | No | **№169** | Yes | Yes | No | No |
| **№50** | Yes | No | Yes | Yes | **№170** | No | No | No | No |
| **№51** | Yes | No | Yes | No | **№171** | Yes | Yes | No | No |
| **№52** | No | No | No | No | **№172** | No | No | No | No |
| **№53** | No | No | No | No | **№173** | Yes | Yes | No | No |
| **№54** | Yes | No | Yes | Yes | **№174** | No | No | No | No |
| **№55** | No | No | No | No | **№175** | Yes | Yes | No | No |
| **№56** | Yes | Yes | No | No | **№176** | No | No | No | No |
| **№57** | No | No | No | No | **№177** | No | No | No | No |
| **№58** | No | No | No | No | **№178** | Yes | Yes | No | No |
| **№59** | Yes | No | Yes | No | **№179** | No | No | No | No |
| **№60** | No | No | No | No | **№180** | Yes | No | Yes | Yes |
| **№61** | Yes | Yes | No | No | **№181** | Yes | Yes | No | No |
| **№62** | Yes | Yes | Yes | Yes | **№182** | Yes | Yes | No | No |
| **№63** | Yes | Yes | Yes | Yes | **№183** | No | No | No | No |
| **№64** | Yes | Yes | Yes | Yes | **№184** | Yes | Yes | No | No |
| **№65** | Yes | Yes | No | No | **№185** | Yes | Yes | No | No |
| **№66** | No | No | No | No | **№186** | Yes | No | Yes | Yes |
| **№67** | Yes | Yes | No | No | **№187** | No | No | No | No |
| **№68** | No | No | No | No | **№188** | Yes | Yes | No | No |
| **№69** | No | No | No | No | **№189** | Yes | Yes | No | No |
| **№70** | Yes | Yes | No | No | **№190** | Yes | Yes | No | No |
| **№71** | No | No | No | No | **№191** | Yes | Yes | No | No |
| **№72** | Yes | No | Yes | No | **№192** | Yes | Yes | No | No |
| **№73** | No | No | No | No | **№193** | No | No | No | No |
| **№74** | Yes | No | Yes | No | **№194** | No | No | No | No |
| **№75** | Yes | No | Yes | No | **№195** | Yes | No | Yes | Yes |
| **№76** | Yes | No | Yes | No | **№196** | Yes | No | Yes | Yes |
| **№77** | No | No | No | No | **№197** | Yes | Yes | No | No |
| **№78** | Yes | No | Yes | No | **№198** | No | No | No | No |
| **№79** | Yes | No | Yes | No | **№199** | Yes | No | No | No |
| **№80** | No | No | No | No | **№200** | Yes | Yes | No | No |
| **№81** | Yes | No | Yes | No | **№201** | Yes | Yes | No | No |
| **№82** | No | No | No | No | **№202** | Yes | Yes | No | No |
| **№83** | No | No | No | No | **№203** | No | No | No | No |
| **№84** | Yes | No | Yes | No | **№204** | No | No | No | No |
| **№85** | Yes | Yes | Yes | Yes | **№205** | Yes | No | Yes | No |
| **№86** | Yes | No | Yes | No | **№206** | No | No | No | No |
| **№87** | No | No | No | No | **№207** | Yes | Yes | No | No |
| **№88** | No | No | No | No | **№208** | Yes | No | No | No |
| **№89** | Yes | No | Yes | No | **№209** | Yes | Yes | No | No |
| **№90** | Yes | No | Yes | No | **№210** | Yes | Yes | No | No |
| **№91** | Yes | No | Yes | No | **№211** | Yes | Yes | No | No |
| **№92** | Yes | No | Yes | No | **№212** | Yes | Yes | No | No |
| **№93** | No | No | No | No | **№213** | Yes | Yes | No | No |
| **№94** | Yes | No | Yes | No | **№214** | Yes | Yes | No | No |
| **№95** | No | No | No | No | **№215** | Yes | Yes | Yes | Yes |
| **№96** | Yes | No | Yes | No | **№216** | Yes | Yes | No | No |
| **№97** | Yes | No | Yes | No | **№217** | Yes | No | Yes | No |
| **№98** | No | No | No | No | **№218** | No | No | No | No |
| **№99** | Yes | No | No | No | **№219** | Yes | No | Yes | Yes |
| **№100** | Yes | No | No | No | **№220** | Yes | No | Yes | No |
| **№101** | Yes | No | No | No | **№221** | Yes | No | Yes | No |
| **№102** | No | No | No | No | **№222** | Yes | No | Yes | No |
| **№103** | Yes | Yes | No | No | **№223** | No | No | No | No |
| **№104** | Yes | No | No | No | **№224** | Yes | No | Yes | No |
| **№105** | Yes | No | Yes | Yes | **№225** | Yes | No | Yes | No |
| **№106** | Yes | No | Yes | Yes | **№226** | Yes | No | No | No |
| **№107** | Yes | No | Yes | No | **№227** | Yes | No | No | No |
| **№108** | Yes | No | Yes | No | **№228** | Yes | Yes | No | No |
| **№109** | Yes | No | Yes | Yes | **№229** | No | No | No | No |
| **№110** | Yes | Yes | No | No | **№230** | Yes | No | Yes | No |
| **№111** | Yes | No | Yes | No | **№231** | No | No | No | No |
| **№112** | Yes | No | Yes | No | **№232** | No | No | No | No |
| **№113** | Yes | Yes | No | No | **№233** | Yes | No | Yes | No |
| **№114** | Yes | No | No | No | **№234** | Yes | Yes | No | No |
| **№115** | No | No | No | No | **№235** | No | No | No | No |
| **№116** | Yes | No | Yes | No | **№236** | No | No | No | No |
| **№117** | No | No | No | No | **№237** | No | No | No | No |
| **№118** | No | No | No | No | **№238** | Yes | No | Yes | No |
| **№119** | No | No | No | No | **№239** | Yes | Yes | No | No |
| **№120** | Yes | No | Yes | No | **№240** | No | No | Yes | No |
